# Liposome Technology for Industrial Purposes

**DOI:** 10.1155/2011/591325

**Published:** 2010-12-05

**Authors:** Andreas Wagner, Karola Vorauer-Uhl

**Affiliations:** ^1^Polymun Scientific Immunbiologische Forschung GmbH, Nu*β*dorfer Lände 11, 1190 Vienna, Austria; ^2^Department of Biotechnology, University of Natural Resources and Applied Life Sciences, Muthgasse 11, 1190 Vienna, Austria

## Abstract

Liposomes, spherical vesicles consisting of one or more phospholipid bilayers, were first described in the mid 60s by Bangham and coworkers. Since then, liposomes have made their way to the market. Today, numerous lab scale but only a few large-scale techniques are available. However, a lot of these methods have serious limitations in terms of entrapment of sensitive molecules due to their exposure to mechanical and/or chemical stress. This paper summarizes exclusively scalable techniques and focuses on strengths, respectively, limitations in respect to industrial applicability. An additional point of view was taken to regulatory requirements concerning liposomal drug formulations based on FDA and EMEA documents.

## 1. Introduction

### 1.1. History, Definition, and Classification of Liposomes

The story of success of liposomes was initiated by Bangham and his colleagues in the early 1960s who observed that smears of egg lecithin reacted with water to form quite intricate structures. They were analyzed by electron microscopy showing that a multitude of vesicles were formed spontaneously. These more or less homogenous lipid vesicles were first called smectic mesophases [1]. Later on, a colleague of Bangham termed them—more euphoniously—liposomes [2]. In the following years, liposomes were primarily used as artificial membrane models mimicking simple cell systems for the investigation of transport functions and mechanisms, permeation properties, as well as adhesion and fusion kinetics. Liposomes were very soon recognized as promising candidates for drug delivery systems [3, 4], and in this regard more and more tailor-made formulations were investigated for certain purposes such as medical applications, cosmetics but also in food and agricultural industry, whereby the main activities were focused on pharmaceutical and in particular biopharmaceutical applications. The first most prominent products are Doxil (Sequus) and DaunoXome (Gilead, Nexstar). Both are indicated as anticancer drugs, which were successfully tested in clinical studies, followed by the US Food and Drug Administration (FDA) approval in the 1990s.

In general, liposomes are defined as spherical vesicles with particle sizes ranging from 30 nm to several micrometers. They consist of one or more lipid bilayers surrounding aqueous compartments, where the polar head groups are oriented towards the interior and exterior aqueous phases. However, self-aggregation of polar lipids is not restricted to conventional bilayer structures which depend on temperature, molecular shape, and environmental and preparation conditions but may self-assemble into various kinds of colloidal particles [5, 6].

Due to this fact, the liposome family includes various kinds of colloidal particles and structures which hamper systematic classification. However, they can be classified by structure, composition, and preparation, as shown in Table 1. 

Technology and application are driven by two major facts. First, the transfer from academic bench to a highly regulated, high technology industry was difficult for liposome technology because of the lack of appropriate methods to produce large quantities in a controlled and reproducible manner. Although several methods are suitable for large-scale production, their development, implementation, and quality control needed a certain time. Second, early clinical trials were not as successful as expected because the stability of conventional liposomes was low, caused by inefficient preparation, physical properties, and unfavorable choice of lipids. Furthermore, they were to a great extent cleared by liver and spleen very rapidly so that neither a prolonged biological half-life nor specific targeting was achieved. More stable conventional liposomes and second-generation formulations, such as the stealth technology, gave new impulses to the industry as well as to clinicians with the development of industrial processes in the 1990s.

### 1.2. Liposome Technology and Regulatory Requirements

In the last decade, the European Agency of the Evaluation of Medical Products (EMA) as well as the FDA has implemented the subject of liposome into their guidelines.

Currently, EMA has not yet published any summarizing document or guideline which is dealing exclusively with nanoparticular structures. However, general aspects of liposomes are covered in several guidelines such as “Note of Guidance on the Quality, Preclinical and Clinical Aspects of gene transfer medicinal,” and “Guideline on adjuvant in vaccines for human use”. 

Regarding appropriate regulations, FDA published a draft version in 2001 entitled “Liposome Drug Products: chemistry, manufacturing, and controls, human pharmacokinetics and bioavailability and labeling documentation.” This draft version includes recommendations explicitly for liposome drug products submitted in new drug applications (NDAs). In detail, recommendations concerning the submission of a new liposomal product are given regarding physiochemical properties, description of manufacturing process and process controls, and control of excipients and drug products. Control of excipients includes all parameters which are necessary to define lipid components, including description, characterization, manufacture, and stability. Control of drug products is dealing with the specifications. In principal, the recommendations of the ICH (International Conference on Harmonization) guideline Q6A “Specifications, Test Procedures and Acceptance criteria for New Drug Substances and New Drug Products: Chemical Substances” are appropriate, but additional testing is necessary. In particular, physicochemical parameters are critical for product quality for each batch. Furthermore, aspects are addressed such as assaying encapsulated and nonencapsulated drug substance, lipid components, and degradation products, as well as *in vitro* tests for drug release from liposomes. The second part of this document is dealing with human pharmacokinetics and bioavailability. In particular, requirements concerning the quality and potency of bioanalytical methods are discussed. Therefore, the recommendations are focused on the validation of these methods and the capability to distinguish between encapsulated and nonencapsulated drug substances. Similar recommendations are given for *in vivo* integrity and stability considerations, respectively. For safety assessment, validated *in vitro *assays are recommended to simulate the liposomal release and/or interaction with lipoproteins and other proteins in the blood. In an additional chapter, studies for pharmacokinetics and bioavailability are recommended, such as mass balance studies and pharmacokinetic studies. Finally, general recommendations concerning the labeling requirements are given. This draft guidance does not provide recommendations on clinical efficacy and safety studies, nonclinical pharmacology and/or toxicology studies, bioequivalence studies or those to document the sameness, liposomal formulations of vaccine adjuvant or biologics, and drug-lipid complexes. Unfortunately, during the intensive discussion process no conclusion regarding the appropriate approaches to access pharmacokinetics and bioavailability was achieved. Hence, this document has only draft status to this date.

In 2006, a reflection paper was published on nanotechnology-based medicinal products for human use reflecting the current thinking and the initiatives by EMA in view of recent developments in relation to this scope. As mentioned in this document, medicinal products containing nanoparticles, including liposomes, have already been authorized both in EU and US under the existing regulatory frameworks. Nevertheless, the European Commission has developed a number of initiatives with emphasis on safety and ethical considerations but also to evaluate the appropriateness of existing methodologies to assess the potential risks associated with nanotechnology. In this context, it is mentioned that there is still insufficient knowledge and data concerning nanoparticles characterization, their detection and measurement, the persistence of nanoparticles in humans and the environment, and all aspects of toxicology related to these particles to allow satisfactory risk assessments. In order to deal with this issue, the EMEA has created the Innovative Task Force for the coordination of scientific and regulatory competence. Because novel applications of nanotechnology will span the regulatory boundaries between medicinal products and medical devices, the mechanism of action will be the key to decide whether a product should be regulated as a medical product or a medical device. Furthermore, evaluation of the quality, safety, efficacy, and risk management must be discussed in more detail. In conclusion, it is likely that the evaluation of such new products will require special considerations. Therefore, EMA will promote this process either to develop specific guidelines or for the update of existing once.

## 2. Preparation Techniques

### 2.1. General Introduction into Techniques

Lipid molecules have to be introduced into an aqueous environment for the preparation of liposomes independent of liposome size and structure. A general overview representing the correlation of the way of lipid hydration, respectively, the way of primary liposome formation with the resulting liposome structure, was originally developed by Lasic [38].

Several ways of treating the lipids are known to support the hydration of these molecules, as lipid molecules themselves are poorly soluble in aqueous compartments. These procedures can be categorized as shown in Table 2.

Additional methods have been developed such as freeze thawing, freeze drying, and extrusion. However, they are all based on preformed vesicles. In the following sections, liposome preparation techniques are described with respect to the principle of lipid hydration/liposome formation as well as process design and description. In addition, the advantages and disadvantages of each technique are pointed out. Furthermore, focus is given on discussing the techniques with respect to their applicability regarding large-scale production for clinical purposes and good manufacturing practice (GMP) relevant issues.

## 3. Mechanical Methods

### 3.1. Preparation by Film Methods

Properties of lipid formulations can vary depending on the composition (cationic, anionic, and neutral lipid species). However, the same preparation method can be used for all lipid vesicles regardless of composition. The general steps of the procedure are preparation of the lipids for hydration, hydration with agitation, and sizing to a homogeneous distribution of vesicles [40]. 

Since then, many different variations of this method have been developed differing in the organic solvents used for lipid solubilization, the way of lipid drying, and the way of film rehydration.

Despite the various modifications, all these methods have in common that heterogeneous populations of multilamellar liposomes are produced. However, vesicle size is influenced by the lipid charge. Charged lipids form smaller liposomes with less lamellae. Other influencing parameters are the nature of the aqueous phase as well as energy and power input of agitation.

The film method has several advantages. It can be used for all different kinds of lipid mixtures. In addition, the method is easy to perform, and high encapsulation rates of lipid as well as aqueous soluble substances can be achieved because high lipid concentrations can be used.

One major drawback of this method is the difficulty of scaling up to several tens of liters. Furthermore, the process becomes more time and cost intensive because additional processing is recommended for a defined liposome suspension, whereby product losses are generated.

Several downsizing techniques have been established in order to make the heterogeneous vesicles more uniform. The first published downsizing method was sonication [41]. A very high energy input based on cavitation is applied to the liposomal dispersion either directly with a tip or indirectly in a bath sonicator.

Other methods also aiming at breaking down the large MLVs are homogenization techniques, either by shear or pressure forces. In this group, methods are included such as microfluidization, high-pressure homogenization, and shear force-induced homogenization techniques.

The most defined method for downsizing is the extrusion technique whereby liposomes are forced through filters with well defined pores.

### 3.2. Homogenization Techniques

Similar to the ultrasound methods, homogenization techniques have been used in biology and microbiology for breaking up the cells. Therefore, many scientists have used them for reducing the size and number of lamellae of multilamellar liposomes.

The French press [42] originally was established for breaking up cells under milder and more appropriate conditions compared to the ultrasound techniques, because lipids as well as proteins or other sensitive compounds might be degraded during the sonication procedure. This system is normally used in the volume of 1 to 40 mL and therefore is not suitable for large-scale production. However, a scale-up-based strategy on this technique was established as the microfluidization. This continuous and scaleable variation of the French press technique enforces downsizing of Liposomes by collision of larger vesicles at high pressure in the interaction chamber of the microfluidizer.

Starting volumes from 50 mL upwards are applicable, and again high pressures are used for disruption of multilamellar systems. The system works in a pressure range of 0–200 bar and is equipped with heating and cooling systems to control sample temperature during processing [43]. The liposome suspension passes the exchangeable orifices several times (up to thousands of passes). Liposomes are formed in the size range from 50 to 100 nm by this process. This technique is suitable for large-scale production and sterile liposome preparation.

In contrast to the microfluidizer, where the fluid stream is split and mixed by collision in a mixing chamber, homogenizers work on a different principle. In a homogenizer, the fluid beam is pressed with high pressure through an orifice, and this beam collides with a stainless steel wall. The liposome suspension is continuously pumped through the homogenizer system, where high pressures are generated to downsize lipid vesicles [44].

The most prominent scalable downsizing method is the extrusion. Size reduction is managed under mild and more reproducible conditions compared to those discussed above. In this method, preformed vesicles are forced through defined membranes by a much lower pressure as described in the French press method. Extrusion through polycarbonate filters was first published by Olson et al. in 1979 [45]. Mayer et al. [19] performed extensive studies on varying lipid compositions and the influence on extrusion behavior and membrane properties. Depending on the apparatus and scale, the diameters of these membranes range from 25 to 142 mm. Lipex Biomembranes Inc., now Northern Lipids Inc., invented a vessel system for extrusion which is marketed from the mL scale to several liters. As suggested for all downsizing methods, liposomes should be extruded above the Tc of the lipid composition; this system can be tempered. The Lipex extruder system is available in a jacketed mode to allow extrusion at higher temperatures.

An alternative is the Maximator device, established by Schneider et al. [46]. It is a continuous extrusion device working with a pumping system. The Maximator consists of a thermostable glass supply vessel directly connected to a pneumatic high pressure piston pump. The latter is driven by either oxygen or nitrogen at pressures below 0.5 MPa (5 bar or 75 psi). The propellant gas does not come into contact with the liposome suspension. The resulting operating (extrusion) pressure—which can be adjusted via the reduction valve in the control device for the propellant gas (3)—can be as high as 12 MPa (120 bar or 1800 psi) with the current equipment.

All the presented extrusion methods have in common that the reproducibility of downsizing is extremely high. Systems with a heating device can either be used with saturated and unsaturated lipids and are; therefore, all purpose systems.

The main disadvantage of this method is the long-lasting preparation starting with preformed liposomes, eventually an additional freeze-thaw step, and finally the extrusion. In these entire procedures, high product losses may be generated, especially if clogging of the extrusion membranes occurs, which may cause technical limitations with large-scale production of high-priced goods.

## 4. Methods Based on Replacement of Organic Solvents by Aqueous Media

The liposome preparation methods described in this section have in common that organic solvents, either water miscible or immiscible, are replaced by an aqueous solution. This replacement is either performed by injection of the lipid carrying organic solution into the aqueous phase—the injection methods—or by stepwise addition of aqueous phase to the organic phase, in particular ethanol—the proliposome-liposome method. In addition, the emulsification methods, namely, the reverse-phase evaporation method and the double emulsion technique, are based on the replacement of a water-immiscible solvent by an aqueous phase, thus forming liposomes with high encapsulation rates of hydrophilic as well as lipid phase soluble substances.

### 4.1. The Ethanol Injection Method

This technique was first reported in the early 1970s by Batzri and Korn [47] as one of the first alternatives for the preparation of SUVs without sonication. By the immediate dilution of the ethanol in the aqueous phase, the lipid molecules precipitate and form bilayer planar fragments [48] which themselves form into liposomal systems, thereby encapsulating aqueous phase. Batzri and Korn performed their experiments with a very low lipid concentration resulting in small liposomes and poor encapsulation efficiency.

The preparation parameters influencing liposome size, size distribution, and drug encapsulation efficiency were investigated in more detail by Kremer et al. in 1977 [49]. They determined the lipid concentration in ethanol as the only liposome formation influencing parameter. Neither stirring rate of the aqueous phase nor injection velocity had a significant influence on liposome size and size distribution.

Another modified ethanol injection method was developed by Maitani et al. [50] which is more or less a combination of the ethanol injection method, the proliposome method, and the reverse-phase evaporation technique.

This method has many advantages as the technique is in principle easy to scale up, and ethanol is a very harmless solvent, accepted by the authorities also for injectables at a maximum of 0.1% [51]. Some other solvents might also be used, but one has to keep in mind the regulations for residual solvents classified into different categories by the European or US Pharmacopoeia. Stano et al. [52] emphasize the advantage of preparing monomodal distributed liposomes in the size range of about 100 nm and furthermore point out the suitability of the entrapment of lipophilic substances. An additional advance is the improvement of product shelf life due to the absence of mechanical forces which might lead to drug and/or lipid degradation.

Therefore, further development was initiated by several groups. In 1995, Naeff [53] published the development of a liposome production technique in industrial scale based on the ethanol injection technique. Their production system was used for the liposomal encapsulation of econazole, an imidazole derivative for the topical treatment of dermatomycosis, and combined the principles of the ethanol injection system and high shear homogenization.

Additional production technology patents from several companies were filed dealing with liposome production systems based on the ethanol injection technique (Optime, Liposome Comp. Martin, Tenzel) [54–57].

Wagner et al. have also extensively worked in this field, leading to the development of the cross-flow injection system. Based on the ethanol injection technique, they developed a scalable and sterile production technique leading from the conventional batch process to a continuous procedure [58].

Herein, the principal item is the cross-flow injection module [59], especially designed for this purpose. This specially conceived unit has the benefit of defined and characterized injection streams and permits liposome manufacture regardless of production scale because scale is determined only by free disposable vessel volumes. By this, process development is performed in lab scale at a volume of about 20 mL. Once the parameters are defined, an easy scale-up can be performed by changing the process vessels only. In addition, these process vessels can be sterilized, either by steam or autoclavation. All raw materials such as buffer solutions, lipid ethanol solution, and even N_2_ for applying the injection pressure are transferred into the sanitized and sterilized system via 0.2 *μ*m filters to guarantee an aseptic production [60].

Liposome size can be controlled by the local lipid concentration at the injection point which is defined by the lipid concentration in ethanol, the injection whole diameter, the injection pressure, and the flow rate of the aqueous phase. By varying these parameters, different liposome sizes suitable for the intended purpose can be prepared. These defined process parameters are furthermore responsible for highly reproducible results with respect to vesicle diameters and encapsulation rates [61]. Tangential flow filtration is the next process step to remove ethanol as well as not entrapped drug.

Another important advantage of this method is the suitability of the entrapment of many different drug substances [61] such as large hydrophilic proteins by passive encapsulation, small amphiphilic drugs by a one-step remote loading technique, or membrane association of antigens for vaccines [62].

### 4.2. Proliposome-Liposome Method

The proliposome-liposome method is based on the conversion of the initial proliposome preparation into a liposome dispersion by dilution with an aqueous phase [50]. This method is suitable for the encapsulation of a wide range of drugs with varying solubility in water and alcohol and has extremely high encapsulation efficiencies when compared with other methods based on passive entrapment. Turánek and coworkers [63, 64] have developed a sterile liposome production procedure based on this method. Reproducible liposome preparation is feasible in a controlled manner by exact controlling of the dilution rate and process temperature. Additionally, the authors claim their method as being easy to scale up, which makes this method an alternative approach for the production of liposomes for clinical application.

### 4.3. Reverse-Phase Evaporation (REV)

Similarly to the above presented injection methods, lipid is hydrated via solubilization in an organic phase followed by introduction into an aqueous phase. The organic phase should be immiscible with the aqueous phase, thus an oil/water emulsion is created, which is diluted with further aqueous phase for liposome formation [65]. The advantage of this very popular preparation technique is a very high encapsulation rate up to 50%. One variation of the microemulsion technique, the double emulsion technique, further improves the encapsulation rates and results in unilamellar liposomes [26]. A possible drawback of this efficient method is the remaining solvent or the proof of their absence especially for using them for pharmaceutical purposes. The other important issue is large-scale production which might be feasible if appropriate shear mixing devices for the creation of the microemulsion and pumps for the dilution step are available.

## 5. Methods Based on Detergent Removal

In this group of liposome preparation procedures, detergents, such as bile salts or alkylglycosides, are used for the solubilization of lipids in micellar systems. In contrast to lipids, detergents are highly soluble in both aqueous and organic media. There is equilibrium between the detergent molecules in the aqueous phase and the lipid environment of the micelle. The size and shape of the resulting vesicles are depending on the chemical nature of the detergent, their concentration, and the lipids used. To date, the most frequently applied method for membrane protein reconstitution involves the cosolubilization of membrane proteins and phospholipids [66–68]. Common procedures of detergent removal from the mixed micelles are dilution [69], gel chromatography [70], and dialysis through hollow fibers [71] or through membrane filters [72]. Additionally, detergents can also be removed by adsorption to hydrophobic resins or cyclodextrins [73]. Dialysis of mixed micelles against an aqueous medium was first described by Kagawa and Racker [74]. This method for vesicle formation is based on the retention of the micelle, whereas free detergent molecules are eliminated. Goldin [72] describe the use of pure cellulose for this approach. In order to gain better control in the formation of proteoliposomes, Wagner et al. developed a new technique, where they combine an advanced ethanol injection technique, the cross-flow injection technique [58], with detergent dilution within one operational step. Thereby, lipid vesicles are formed immediately after injection into a micellar protein solution. As described earlier, the multiple injection technique [59], previously used for high yield passive encapsulation of water-soluble proteins, can be adapted for this one-step detergent dilution/vesicle forming process [62].

## 6. Final Remarks

Numerous studies for the pharmaceutical application of liposomes have appeared during the past few decades. They have attracted great interest as models for biological membranes, diagnostics, nutrients, and other bioactive agents. Nevertheless, the pharmaceutical application, as drug carriers for specific targeting, controlled, and/or sustained release, as well as for vaccination, was and still is the driving force for the development of innovative technologies. From this expertise, one can derive that liposomes are versatile carrier systems which need to be custom made in terms of *in vitro* and *in vivo* properties. In the last decades, numerous preparation techniques were established for this purpose, whereby most of them are in particular suitable for the laboratory and less for industrial approach. However, large scale capacities are required for the preparation of clinical material as well as for marketed products providing sterile, well-characterized, and stable products. Unfortunately, the availability of certain production methods as well as the quality aspects depend on the characteristics of the lipids themselves. This limits the choice of liposome types from which one can select when optimizing liposome-based drug therapy.

Though many preparation methods were investigated in the 1980s and 1990s, little attention has been paid to the transfer of technology to industry. Thus, presently the advancement is primarily focused on large-scale manufacturing. Stringent control of the product is required to ensure the predictable therapeutic effect, whereas acceptance criteria have to be defined for the quality as well as the process. Additionally, quality issues regarding unwanted by-products, such as residues of organic solvents and/or degradation products, are just as important as pyrogen-free and sterile conditions. In particular, the latter aspect still is a big issue for industrial processes. Until now, no general acceptable method could be successfully established. Commonly used processes to achieve sterility for pharmaceutical products are sterile filtration or autoclaving. Both methods are of either no or only limited suitability for liposomal drug products. In many cases, degradation and/or unacceptable product loss in combination with drug release and instability are the consequences. Currently, many manufacturers try to implement alternative strategies, such as lyophilization and production processes in closed containments equipped with sterile filter barriers, to solve this essential problem.

In conclusion, besides the development of new liposomal drug formulations, researchers as well as manufacturers are required to establish processes which are state of the art in the pharmaceutical industry. The realization of maybe future, more complex liposome structures with advanced efficacy will to a great extent dependent on those achievements.

## Figures and Tables

**Table 1:** Classification of commonly known lipid vesicles according to their structures and/or preparation.

Identification	Definition
Archeosomes	Archeosomes are vesicles consisting of archebacteria lipids which are chemically distinct from eukariotic and prokariotic species. They are less sensitive to oxidative stress, high temperature, and alkaline pH [7, 8].

Cochleates	Cochleates are derived from liposomes which are suspended in an aqueous two-phase polymer solution, allowing the logic partitioning of polar molecule-based structures by phase separation. The liposome containing two-phase polymer solution treated with positively charged molecules such as Ca^2+^ or Zn^2+^ forms a cochleate precipitate of a particle dimension less than 1 *μ*m [9].

Dendrosomes	Dendrosomes represent a family of novel, nontoxic, neutral, biodegradable, covalent or self-assembled, hyperbranched, dendritic, spheroidal nanoparticles which are easy to prepare, inexpensive, highly stable as well as easy to handle and apply, compared with other existing synthetic vehicles for gene delivery [10].

Dried reconstituted vesicles (DRV)	By this preparation technique, small, “empty” unilamellar vesicles, containing different lipids or mixtures of them, are prepared. After mixing those SUVs with the solubilised drug, dehydration is performed. By addition of water, rehydration leads to the formation of large quantities of rather inhomogeneous, multilamellar vesicles which need further processing [11].

Ethosomes	Ethosomal systems are much more efficient at delivering to the skin, in terms of quantity and depth, than either conventional liposomes or hydroalcoholic solutions. Ethosomal drug permeation through the skin was demonstrated in diffusion cell experiments. Ethosomal systems composed of soy phosphatidylcholine and about 30% of ethanol were shown to contain multilamellar vesicles by electron microscopy [12].

Immunoliposomes	Liposomes modified with antibodies, Fab's, or peptide structures on the bilayer surface were established for *in vitro* and *in vivo* application [13, 14].

Immunosomes	Immunosomes are prepared by the anchorage of glycoprotein molecules to preformed liposomes. Under the electron microscope, immunosomes look like homogenous spherical vesicles (50–60 nm) evenly covered with spikes. Immunosomes have structural and immunogen characteristics closer to those of purified and inactivated viruses than any other form of glycoprotein lipids association [15].

Immune stimulating complex (ISCOM)	ISCOMs are spherical, micellar assemblies of about 40 nm. They are made of the saponin mixture Quil A, cholesterol, and phospholipids. They contain amphiphilic antigens like membrane proteins. ISCOMs already have a built-in adjuvant, *Quillaja *saponin, which is a structural part of the vehicle [16].

Lipoplexes	Cationic lipid-DNA complexes, named lipoplexes, are efficient carriers for cell transfection but have certain drawbacks due to their toxicity. These toxic effects may result from either cationic lipids or nucleic acids [17, 18].

LUVETs	LUVETs are large unilamellar vesicles prepared by extrusion technique, mainly performed with high-pressure systems [19].

Niosomes	Niosomes are small unilamellar vesicles made from nonionic surfactants also called Novasomes. Their chemical stability is comparable to that of archeosomes [20].

pH-sensitive liposomes	Four basic classes of pH-sensitive liposomes have been described previously. The first class combines polymorphic lipids, such as unsaturated phosphatidylethanolamines, with mild acidic amphiphiles that act as stabilizers at neutral pH. This class of pH-sensitive liposomes has been the most intensively studied. The second class includes liposomes composed of lipid derivatives resulting in increased permeability to encapsulated solutes. A third class of pH-sensitive liposomes utilizes pH-sensitive peptides or reconstituted fusion proteins to destabilize membranes at low pH. The final and most current class of pH-sensitive liposomes uses pH-titratable polymers to destabilize membranes following change of the polymer conformation at low pH [21].

Polymerised liposomes	Polymerized phosphatidyl choline vesicles (35–140 nm) have been synthesized from lipids bearing one or two methacrylate groups per monomer. Compared to nonpolymeric analogues, these vesicles exhibited improved stability and controllable time-release properties [22].

Proliposomes	Proliposomes are defined as dry, free-flowing particles that immediately form a liposomal dispersion on contact with water [23, 24].

Proteosomes	Vesicles of bacterial origin were solubilised, followed by ammonium sulphate precipitation and dialysis against detergent buffer. Proteins and peptides are noncovalently complexed to the membrane, making them highly immunogenic [25].

Reverse-phase evaporation vesicles (REV)	Vesicles are formed by evaporation of oil in water emulsions resulting in large unilamellar liposomes [26].

Stealth liposomes	In the early 1990s, this liposome engineering process culminated with the observation that coating of liposomes with polyethylene glycol (PEG), a synthetic hydrophilic polymer, would improve their stability and lengthens their half-lives in circulation, rendering the use of glycolipids obsolete. PEG coating inhibits protein adsorption and opsonization of liposomes, thereby avoiding or retarding liposome recognition by the reticuloendothelial system (RES). These PEG-coated liposomes are also referred to as sterically stabilized or stealth liposomes. The PEG stabilizing effect results from local surface concentration of highly hydrated groups that sterically inhibit both hydrophobic and electrostatic interactions of a variety of blood components at the liposome surface [27–33].
Temperature-sensitive liposomes	Temperature-sensitive liposomes are considered to be a promising tool to achieve site-specific delivery of drugs. Such liposomes have been prepared using lipids which undergo a gel-to-liquid crystalline phase transition a few degrees above physiological temperature. However, temperature sensitization of liposomes has been attempted using thermosensitive polymers. So far, functional liposomes have been developed according to this strategy whose content release behavior, surface properties, and affinity to cell surface can be controlled in a temperature-dependent manner [34, 35].

Transfersomes	Transfersomes consist of phosphatidylcholine and cholate and are ultradeformable vesicles with enhanced skin-penetrating properties [36].

Virosomes	Virosomes are small unilamellar vesicles containing influenza hemagglutinin, by which they became fusogenic with endocytic membranes. Coincorporation of other membrane antigens induces enhanced immune responses [37].

**Table 2:** Methods of liposome preparation and the resulting product. Partly from Lasic and Barenholz [39].

Method	Vesicles
*Mechanical methods*	
Vortex or hand shaking of phospholipid dispersions	MLV
Extrusion through polycarbonate filters at low or medium pressure	OLV, LUV
Extrusion through a French press cell “Microfluidizer” technique	Mainly SUV
High-pressure homogenization	Mainly SUV
Ultrasonic irritation	SUV of minimal size
Bubbling of gas	BSV

*Methods based on replacement of organic solvent(s) by aqueous media*	
Removal of organic solvent(s)	MLV, OLV, SUV
Use of water-immiscible solvents: ether and petroleum	MLV, OLV, LUV
Ethanol injection method	LUV
Ether infusion (solvent vaporization)	LUV, OLV, MLV
Reverse-phase evaporation	

*Methods based on detergent removal*	
Gel exclusion chromatography	SUV
“Slow” dialysis	LUV, OLV, MLV
Fast dilution	LUV, OLV
Other related techniques	MLV, OLV, LUV, SUV
